# *Agrobacterium tumefaciens*-Mediated Transformation of *Fonsecaea monophora* and *Fonsecaea erecta* for Host-Environment Interaction Studies

**DOI:** 10.3390/jof6040325

**Published:** 2020-11-30

**Authors:** Cristina Isabel Ferrer Villena, Renata Rodrigues Gomes, Larissa Fernandes, Camille Silva Florencio, Amanda Bombassaro, Maria Eduarda Grisolia, Edvaldo da Silva Trindade, Sybren de Hoog, Vania Aparecida Vicente

**Affiliations:** 1 Engineering Bioprocess and Biotechnology Graduate Program, Department of Bioprocess Engineering and Biotechnology, Federal University of Paraná, Curitiba 81530-000, Brazil; ferrercristina06@gmail.com (C.I.F.V.); eduarda.grisolia@ufpr.br (M.E.G.); 2 Microbiology, Parasitology and Pathology Graduate Program, Department of Pathology, Federal University of Paraná, Curitiba 81530-000, Brazil; rrgrenata@gmail.com (R.R.G.); amandabssaro@gmail.com (A.B.); 3 Microbial Biology Graduate Program, Department of Cell Biology, University of Brasília, Brasilia 70910-900, Brazil; lfernandesmatos@gmail.com (L.F.); camille.florencio@yahoo.com.br (C.S.F.); 4 Department of Medical Microbiology, Center of Expertise in Mycology of Radboud University Medical Center/Canisius Wilhelmina Hospital, 6525GA Nijmegen, The Netherlands; 5 Department of Cell Biology, Federal University of Paraná, Curitiba 81530-000, Brazil; edstrindad@gmail.com

**Keywords:** *Fonsecaea*, chromoblastomycosis, *Agrobacterium*, transformation, environmental interaction

## Abstract

The fungal genus *Fonsecaea* contains etiological agents of human chromoblastomycosis, a (sub)tropical, (sub)cutaneous implantation disease caused by plant contact. The invasive potential differs significantly between species. Infections by *Fonsecaea monophora* are believed to originate from the environment and the species has been reported as one of the main causative agents of the disease, but also of cases of primary brain infection. The epidemiology of the disease has not been fully elucidated and questions related to its infection route and virulence are still to be clarified. The environmental species *Fonsecaea erecta* was isolated from organic material and living plants in endemic areas for chromoblastomycosis in Brazil. The present paper describes *Agrobacterium* *tumefaciens*-mediated transformation (AMT) of the environmental species *F. erecta* and the pathogenic species *F. monophora*. We propose the use of *Agrobacterium* transformation for future gene function studies related to *Fonsecaea* virulence and pathogenicity. We evaluated the co-cultivation ratios 1:1, 10:1 and 100:1 (*Agrobacterium*:conidia) at 28 °C during 72 h. pAD1625 and pCAMDsRed plasmids were inserted into both species. Confirmation of transformation was realized by *hph* gene amplification and Southern blot determined the amount of foreign DNA integrated into the genome. In order to evaluate a potential link between environmental and clinical strains, we obtained red fluorescent transformants after pCAMDsRed insertion. We observed by confocal fluorescence microscopy that both *F. monophora* and *F. erecta* were able to colonize the palm *Bactris gasipaes*, penetrating the epidermis. These results contribute to understanding the ability of *Fonsecaea* species to adapt to different environmental and host conditions.

## 1. Introduction

Chromoblastomycosis is a neglected occupational disease [1] caused by several dematiaceous fungi. The infection is chronic and involves cutaneous and subcutaneous tissues [2,3,4,5,6,7,8,9]. Late stages of the infection lead to formation of slow-growing, verrucous or tumor-like ulcerative eruptions [2,10,11,12,13,14,15]. Chromoblastomycosis may be acquired by inoculation of pathogenic agents following penetrating trauma [9]; however, the infection process and the origin of the disease are still not entirely clear [7,16,17,18].

*Fonsecaea* species and their relatives are asexual Ascomycetes belonging to the family Herpotrichiellaceae (order Chaetothyriales) containing numerous agents of human infection and with a significant predisposition to grow in human tissue [16,19]. *Fonsecaea* comprises cryptic species (*Fonsecaea pedrosoi*, *Fonsecaea pugnacius*, *Fonsecaea monophora* and *Fonsecaea nubica*) potentially causing disease [12,15], but the invasive potential differs significantly between species [20,21,22]. *Fonsecaea pedrosoi* and *F. nubica* are narrowly associated with chromoblastomycosis, while *F. monophora* is also involved in primary phaeohyphomycosis of the brain, and occasionally of other organs [12,23,24]. Recently, *F. pugnacius* was reported to develop chromoblastomycosis and subsequently disseminating to the brain in the same host [7].

An effective way to study mechanisms which these pathogens use to infect and develop the disease is to disrupt their genes in a targeted or random manner, obtaining mutants with altered virulence [25]. This represents one of the most frequently used tools to understand the molecular basis of virulence and host specificity of pathogens. Transformation mediated by *Agrobacterium* (AMT) is an excellent method for fungal transformation, because it has a high efficiency, usually with a single copy integrated in the DNA, does not require special equipment, and the experiment material can variably be yeast cells, mycelial fragments, or conidia [26,27]. Selective markers such as hygromycin B resistance can be used for construction of cassettes to delete one or more genes. Fluorescent reporter genes are powerful tools to signal the presence of fungi and their visualization inside host tissue, and monitor morphological alterations related to host–fungal interaction [25].

The present study aimed to transform *Fonsecaea* sibling species mediated by *Agrobacterium tumefaciens* to understand the environmental part of the life cycle, using the native palm *Bactris gasipaes* as a model. We describe AMT of *F. erecta,* an environmental species isolated from a living plant, and the pathogenic species *F. monophora*, transformed with pCAMDsRed and pAD1625. Our study may contribute to reveal the link between environmental and clinical strains, in order to understand how environmental species are in the process of evolution and have eliminated the ability to infect the host.

## 2. Materials and Methods

### 2.1. Strains, Plasmids, and Growth Conditions

Strains used were *Fonsecaea erecta* (CBS 125763) isolated from living plant and the pathogenic species *F. monophora* (CBS 269.37) provided by “Microbiological Collections of the Paranaense Network” (CMRP) at the University Federal of Paraná, Curitiba, Paraná, Brazil. Strains were grown on Sabouraud glucose agar (SGA), pH 5.6. We used *Agrobacterium tumefaciens* strain EHA 105, with (1) binary vector pCAMDsRed (DsRed-Express) which is composed of *DsRed* (reporter gene), hygromycin B resistance (hph) and kanamycin resistance gene for selection in bacteria [28,29]; (2) binary vector pAD1625 which has hygromycin B resistance and ampicillin gene [30,31]. *Agrobacterium tumefaciens* was grown at 28 °C in Luria–Bertani (LB) medium supplemented with 100 µg/mL kanamycin (pCAMDsRed) or 100 µg/mL ampicillin (pAD1625). The strains *F. pedrosoi* CBS 271.37 and *F. pedrosoi* hygromycin B resistance-carrying gGFP plasmid were used as controls [31].

### 2.2. Fonsecaea monophora CBS 269.37 and F. erecta CBS 125763 Susceptibility to the Dominant Selective Markers

For evaluation of the susceptibility of hygromycin B, fungi were inoculated as single points on SGA supplemented with different concentrations of hygromycin B (25, 50, 75, 100 and 150 µg/mL). Test was performed in duplicate. Plates were incubated at 28 °C and evaluated at 3, 7, 14, 21 and 30 days after inoculation. Sensitivity to hygromycin B was estimated based on mycelial growth [32]. As controls, we used *F. pedrosoi* CBS 271.37 and *F. pedrosoi* hygromycin B resistance-carrying gGFP plasmid [31].

### 2.3. Fonsecaea Siblings with A. tumefaciens-Mediated Transformation

*Agrobacterium*-mediated transformation was performed according to Florencio et al. (2018) [31] with few modifications. Conidia of *F. monophora* and *F. erecta* were inoculated in potato dextrose broth (PDB) with chloramphenicol (25 µg/mL), grown for 7 days at 28 °C at 150 rpm, centrifuged and resuspended in induction medium (IM) plus 40 mM of (2-N-morpholine)-ethane sulfonic acid (MES) and 0.2 mM of 3′,5′-dimethoxy-4′-hydroxyacetophenone (AS). *A. tumefaciens* was grown overnight in LB liquid medium supplemented for kanamycin (pCAM-DsRed plasmid) and ampicillin (pAD1625 plasmid), at 28 °C at 200 rpm. The bacteria were centrifuged, washed in saline solution (0.9% NaCl), resuspended in 10 mL of IM+MES+AS and grown for approximately 7 h at 28 °C/150 rpm until reaching a density 0.5 to 0.8 at OD_600nm_. For co-cultivation, yeast cells of *F. monophora* and *F. erecta* were mixed with *A*. *tumefaciens* cells at varying ratios (1:1, 10:1, 100:1). The cell mixtures were plated on induction medium for 3 days at 28 °C. After co-cultivation, cells were scraped off, washed with saline and inoculated on SGA plus 100 µg/mL hygromycin B and cefotaxime, and incubated at 28 °C until appearance of colonies.

### 2.4. Mitotic Stability of the Transformed Colonies

Mitotic stability of random colonies resistant to hygromycin B was established by single-point inoculation on SGA at 28 °C for 1 week. Subsequently, isolates were transferred another 4 times, and then grown on SGA supplemented with hygromycin B for evaluation of maintenance of the resistance marker.

### 2.5. PCR Assay and DNA Hybridization

Detection of the *hph* insertion was performed using four randomly selected transformants of each species (*F. erecta* and *F. monophora)* containing the two different plasmids (pCAMDsRed and pAD1625). Total DNA was extracted according to the method described by Vicente et al. (2008) [17]. Amplification reactions of transformants were performed using primers hph1 (5′AGCGTCTCCGACCTGATG3′) and hph2 (5′CGACGGACGCACTGACGG3′), according to Malonek and Meinhardt [33]. The wild-type strains were used as negative control and the transformed strain with gGFP [31] as positive control. To estimate the number of *hph* integrations on selected transformants, 38 µg of genomic DNA previously digested with *Bgl*II (NEB) was subjected separately to 1% agarose gel electrophoresis, and capillary transferred to Hybrond N+ (GE) membrane. *Bgl*II is a single cutter on T-DNA of pAD1625 and pCAMdsRED. The probe in the hybridization procedure was 0.62 kb of *hph* labeled with digoxigenin (PCR DIG Probe Synthesis Kit, Roche). After hybridization, the membrane was washed (Dig Wash and Block Buffer Set, Roche), and detected with alkaline phosphatase and CDP-Star conjugated anti-digoxigenin Fab antibody (CDP-Star Detection Reagent, Roche, Diagnostics GmbH, Mannheim, Germany). The signal was captured by ImageQuant LAS 4000 (GE Healthcare Bio-Sciences AB GE, Uppsala, Sweden) equipment. Genomic DNAs of *F. erecta* and *F. monophora* were used as negative controls (wild type), while pAN7.1 linearized with *HindI*II was the positive control.

### 2.6. Fluorescence Evaluation of Transformants Carrying DsRed

The fluorescence evaluation was done based on the previous described by Eckert, M. et al. [28]. Eight transformants (four strains of *F. monophora* and four of *F. erecta*) were randomly selected and cultured on SGA with 100 µg/mL hygromycin B and were incubated at 28 °C for 7 days. The red fluorescence of DsRed was detected using a laser scanning confocal microscope A1RSiMP (NIKON, Tokyo, Japan) with the following settings: 561 nm laser for excitation and 575–625 nm filter for emission. The autofluorescence was detected with the following settings: 405 nm laser for excitation/425–475 nm filter for emission of blue fluorescence, 488 nm laser for excitation/500–550 nm for emission of green fluorescence. Images were saved and processed using NIS-Elements Viewer 4.20 software (NIKON). The wild-type strains were used as negative controls.

### 2.7. Inoculation in Bactris Gasipaes

*Bactris gasipaes* was provided by the Brazilian Agricultural Research Corporation EMBRAPA. Plants were grown in vitro in MS medium [34] and inoculated with 10 µL of physiological saline solution containing transformed cells or wild-type strains (negative control) at a concentration of 10^2^ conidia/mL for each species (*F. monophora* and *F. erecta*). The experiments were performed in duplicate per group. Inocula were applied as suspensions around plant roots [35]. Plants were incubated at room temperature and the infection was monitored by Nikon confocal microscopy.

### 2.8. Statistical Analysis

Analysis of variance (ANOVA) of the results was performed; when the F test was significant, subsequent comparisons between different ratios were made using Tukey’s test. SPSS Software (IBM Corp., Armonk, NY, USA) was used for statistical analysis.

## 3. Results

### 3.1. Susceptibility to the Dominant Selective Marker

Susceptibility of *F. monophora* (CBS 269.37) and *F. erecta* (CBS 125763) to hygromycin B was tested prior to transformation assays. Residual growth of *F. monophora* and *F. erecta* at concentrations of 25 µg/mL was observed and the strains were completely inhibited at concentrations of 50, 100 and 150 µg/mL. The MIC concentration was determined as 25 µg/mL of hygromycin, but 100 µg/mL was the concentration which was used for the selection of transformants mediated by *A. tumefaciens*.

### 3.2. Fonsecaea Siblings A. tumefaciens-Mediated Transformation

**Figure 1:**
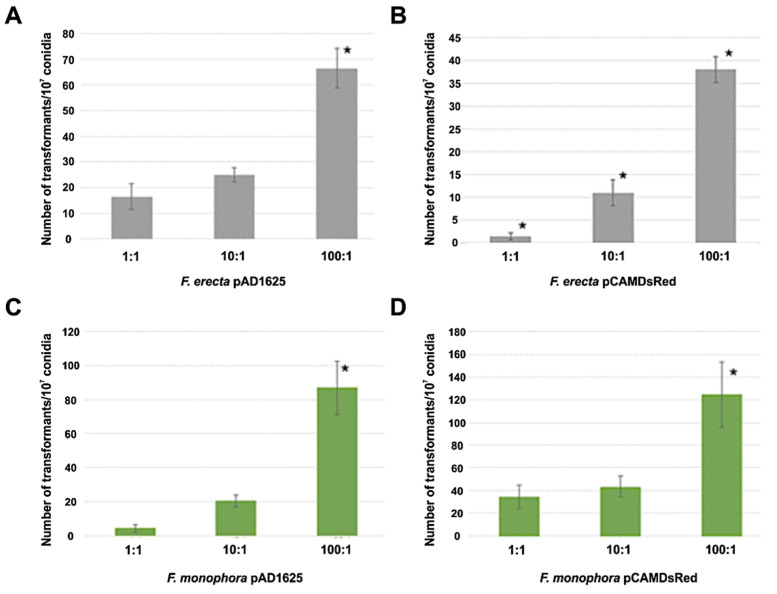
Number of transformants per 10^7^ conidia obtained by AMT. Comparison of transformation efficiency between 1:1, 10:1 and 100:1 (bacteria:conidia) ratios of *Fonsecaea erecta* with pAD1625 (**A**) and pCAMDsRed (**B**), and *Fonsecaea monophora* with pAD1625 (**C**) and pCAMDsRed (**D**) plasmids. The statistical tests applied were one-way ANOVA and Tukey’s post-test. * indicates significant statistical difference with 95% confidence.

Clinical *F. monophora* and environmental *F. erecta* were transformed by *Agrobacterium* with the pAD1625 and pCAMDsRed plasmids. Figure 1 shows the number of transformants per 10^7^ conidia for both plasmids and both species tested after 72 h of co-cultivation at ratios bacteria:conidia of 1:1, 10:1, and 100:1. The AMT with EHA105 harboring pAD1625 with *F. erecta* provided the highest number of transformants (statistically significant) with respect to the pCAMDsRed plasmid. However, for the *F. monophora*, the number of transformants with pCAMDsRed showed a statistical increase compared to pAD1625. The optimal of transformation efficiency was obtained with the ratio bacteria: conidia of 100:1 (Figure 1). All transformants tested were mitotically stable for resistance to hygromycin B at a concentration of 100 µg/mL after five passages in non-selective medium. The transformants conserved the parental morphology of velvet-melanized colonies on SGA plates.

### 3.3. PCR and DNA Hybridization

**Figure 2:**
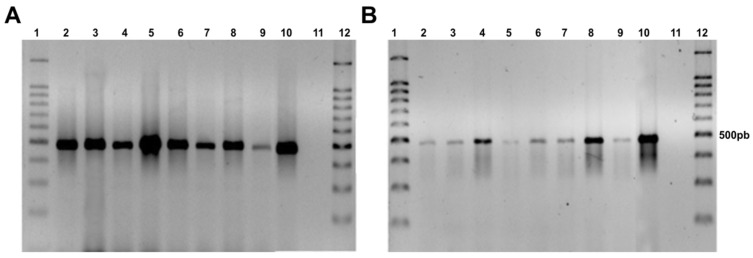
Agarose gel electrophoresis showing presence of the hph gene in transformed colonies with pAD1625 (**A**) and pCAMDsRed (**B**). *Fonsecaea erecta* (2–5), *Fonsecaea monophora* (6–9), *Fonsecaea pedrosoi* gGFP as a positive control (10), *F. monophora* wild type (11), and 100 bp ladder (1,12).

The 500 bp fragment of the *hph* gene was amplified by PCR, demonstrating the presence of the hygromycin B resistance gene in the genome of the *F. erecta* and *F. monophora* transformants, while the wild-type strain did not show any amplification (Figure 2).

**Figure 3:**
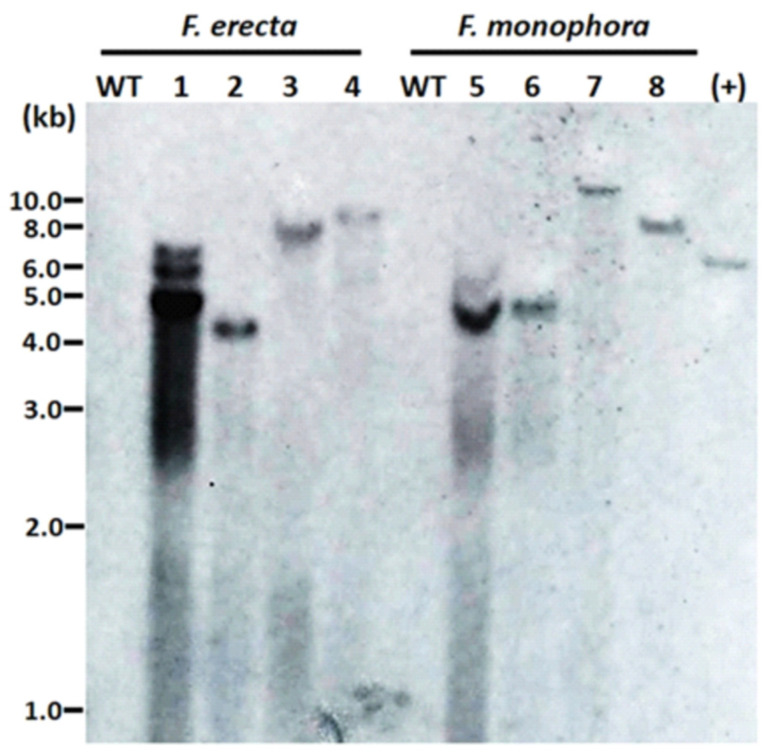
Integrations of T-DNA containing the *hph* gene into the genome of the *Fonsecaea erecta* and *Fonsecaea monophora* after *Agrobacterium*-mediated transformation with pAD1625 (1, 2, 5, 6) or pCAMdsRED (3, 4, 7, 8). A total of 38 μg of genomic DNA was digested with BglII, and 0.6 kb digoxigenin-labelled hph was used as probe. The genomic DNA from the wild-type strain of *F. erecta* (CBS 125763) and *F. monophora* (CBS 269.37) were used as the untransformed negative control (WT), while the HindIII linearized pAN7.1 was the positive control.

The number of T-DNA copies inserted in the genome of the transformants was evaluated by DNA hybridization. Different sites of integration were observed due to size differences of hybridized fragments. In the majority of transformants, we observed single bands, confirming the insertion of a single copy of T-DNA. In one of the transformants, three bands were observed (Figure 3).

### 3.4. Reporter Fluorescence Emission by Fonsecaea Transformants

**Figure 4:**
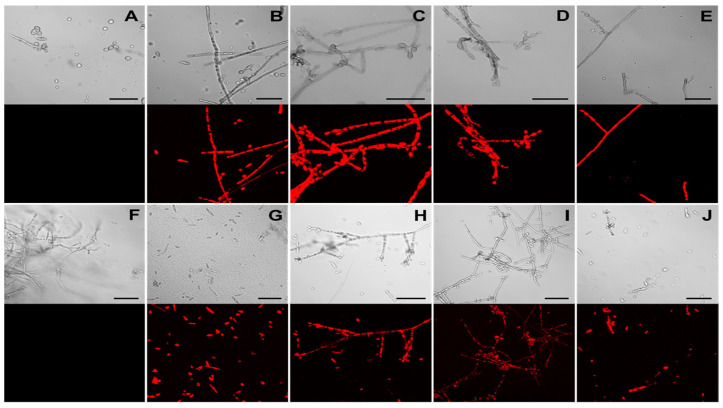
Detection of DsRed fluorescence in *Fonsecaea erecta* (**A**–**E**) and *Fonsecaea monophora* (**F**–**J**) by confocal microscopy. Images of wild-type (**A**,**F**) and transformed strains *F. erecta* 1-pCAMDsRed (**B**), 2-pCAMDsRed (**C**), 3-pCAMDsRed (**D**) and 4-pCAMDsRed (**E**); *F. monophora* 1-pCAMDsRed (**G**), 2-pCAMDsRed (**H**), 3-pCAMDsRed (**I**) and 4-pCAMDsRed (**J**) are shown. The upper panels depict DIC (Differential Interference Contrast) images and the red emitted fluorescence panels are on the bottom.

The presence of the functional DsRed protein in *F. erecta* and *F. monophora* indicating the pCAMDsRed transformed colonies were observed using fluorescent microscopy. Transformed strains exhibited red fluorescence in the cytoplasm of hyphae and conidia, while no red emission was detected in the wild-type strains (Figure 4A,B).

**Figure 5:**
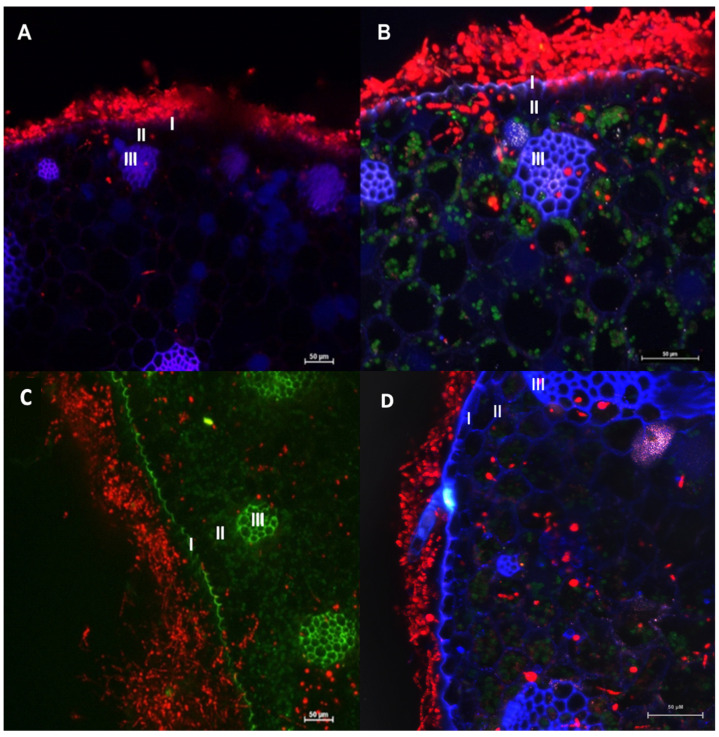
Confocal microscopy images of *Bactris gasipaes* plants inoculated with transformed *Fonsecaea erecta* (**A**,**B**) and *Fonsecaea monophora* (**C**,**D**) expressing DsRed gene (red fluorescence). Blue and green are autofluorescence of plant. It is possible to identify epidermis (I), parenchyma (II) and vascular tissue (III). Green autofluorescence reveals the chloroplasts in B and D.

The transformants of *F. erecta* (4-pCAMDsRed) and *F. monophora* (4-pCAMDsRed) characterized by red fluorescent were inoculated into the model plant *B. gasipaes,* with the wild-type strains as control. *Fonsecaea erecta* and *F. monophora* expressed red fluorescent proteins while colonizing the intercellular spaces (Figure 5).

Moreover, conidia, germinated cells and hyphae were observed on the surface of the epidermis, and in parenchyma and vascular tissue of the plant in Figure 5. Both clinical and environmental strains demonstrated ability to germinate and invade the plant tissues.

## 4. Discussion

Chromoblastomycosis (CMB) is a neglected tropical disease (NTD) caused by dematiaceous fungi and characterized by the presence of muriform cells in tissue, in contrast to phaeohyphomycosis that presents with hyphae. The onset of infection is traumatic inoculation of the fungi from an environmental source [1]. Melanized fungi can be isolated from a wide diversity of natural sources, but the environmental habitat of the clinical species has rarely been revealed. Clinical *Fonsecaea* species have occasionally been detected on plant material [16,36]. *Fonsecaea monophora* and *F. pedrosoi* are generally isolated from clinical samples, while *F. erecta* and *F. minima* are saprobes which have as yet not been found in CBM lesions [16].

The present study demonstrated the feasibility of an *Agrobacterium tumefaciens*-mediated genetic transformation system for *F. erecta* and *F. monophora.* AMT is a powerful tool as it has high transformation frequencies, does not require special equipment, and the transformed cells usually receive a single copy of DNA [26]. The variables used for AMT in the present paper were modified according to Florencio et al. [31], who described a high frequency of transformants using 10^8^ rather than 10^6^ conidia, and a co-cultivation period of 72 instead of 48 h. We used 10^7^ conidia and 72 h of co-cultivation. In addition, we employed *A. tumefaciens* EHA105 as this clone was constructed as a high-virulent strain and was proven to perform excellently in producing high numbers of transformants [37].

*Fonsecaea erecta* and *F. monophora* were completely inhibited by hygromycin B at concentrations up to 50 µg/mL of the antibiotic, confirming data of Florencio et al. [31] for *F. pedrosoi.* This confirms that hygromycin B resistance is an excellent dominant selective marker for genetic transformation of these fungi. A close relationship between conidia ratios and the frequency of *Fonsecaea* transformants was found. Changing the ratio *Agrobacterium*:conidia for both *Fonsecaea* species carrying any of the plasmids (pAD1625 and pCAMDsRed) from 1:1 to 100:1 led to an increase of 4–25 fold in transformation efficiency. The 100:1 ratio generated the highest number of transformants. Florencio et al. [31] used another conidial density, but they also reported an increase in transformation efficiency when a higher conidial ratio was used. Abuodeh et al. [30] reported similar results with *Coccidioides immitis* and Xiao et al. [38] noted that the transformation also increased in the ratio of 100:1 to transform *F. monophora* conidia using *A. tumefaciens* strains AGL-1, although they observed that in EHA105 strains, the highest transformation efficiency was a 1:1 ratio.

The *F. erecta* and *F. monophora* transformants were stable after five rounds of growth. Several investigations described AMT transformants to be mitotically very stable [39,40]. The marker gene (hph) allowed proper selection of transformed strains. Confirmation of hph integration into the genome was performed using PCR and DNA hybridization. The evaluated transformants were amplified using hph specific primers, and Southern blot showed that just a single transformant had three randomly inserted copies of *hph*, while the remaining transformants presented only one hybridized fragment. Michielse et al. [41] also reported that AMT of T-DNA usually inserts a single copy into the fungal genome, with a higher level of mitotic stability of the transformants. Other previous published works also used the same strategy with probes specific to the inserted gene, that in this case was the HPH [27,31,42].

The reporter genes codifying fluorescent proteins can be employed as diagnostic tools in determining the presence of fungal cells in plant hosts [26]. Markers such as GFP and DsRed are widely used because they do not require cofactors or substrates [43], have low toxicity and can be visualized in vivo in individual cells and in cell populations [44]. The DsRed protein (pCAMDsRed) was microscopically detected in hyphae and conidia of all evaluated *Fonsecaea* transformants at a high intensity emission. Eckert et al. [29] noted higher fluorescence emission of DsRed protein than GFP protein in mycelium of *Leptosphaeria* spp. and *Oculimacula* spp.; this may be attributed to the strength and compatibility of the promoter (PgpdA) controlling the reporter genes.

*Bactris gasipaes* is a palm native to the tropical forests reported as the habitat of melanized fungi [45]. Based on this fact, and wondering how *Fonsecaea* species can use the plant as a natural reservoir, we inoculated *B. gasipaes* with *F. erecta* and *F. monophora* transformed conidial cells expressing red fluorescent protein. The inoculum in vitro was applied around the root according to Fornari et al. [35], and both clinical and environmental strains were observed in the root on the location of application of the inoculum and also in the tissues of the stem indicating the ability of invasion, colonization and adaptation of these species to living plant tissue.

Judging from the expression of transformed fungal cells of both species inside the plant tissues, it was observed that transformed strains were able to invade the epidermis, parenchyma and vascular and grow inside tissue of *B. gasipaes* (Figure 5). However, in this study, we observed that the clinical species *F. monophora* was also able to grow in the vascular tissue. Chromoblastomycosis is characterized by the presence of muriform cells inside host tissues. In these studies, as well as in Fornari et al. [36], the cells were not observed inside the plant tissues. However, de Hoog et al. [45] demonstrated that in *Cladophialophora* both the clinical species, *C. carrionii*, and the environmental species, *C. yegresii* could produce muriform cells upon their artificial inoculation into cactus plants.

Since the *A. tumefaciens*-mediated transformation technique is used for random insertional mutagenesis, it is important to assess whether the biological function has been maintained. We observed that the transformed isolates kept their ability to invade plant tissues since they remained able to penetrate the epidermis, reaching the cortical region and clearly growing in the intercellular spaces that were colonized by the fungus. Therefore, the clinical and environmental *Fonsecaea* strains obtained in this study could be used for research in the future.

Our results highlight that both species, *F. monophora* and *F. erecta*, have the ability to penetrate plant epidermis and deeper tissue of our model plant, where we found the transformants expressing DsRed in the area that generates the thorns, which have been the hypothesized transmission route of *Fonsecaea* from the environment to the human host. Our study proposes a new protocol allowing observation of *Fonsecaea* sp. inside plants without histological staining. This approach enables evaluation of the fungi in living tissue. This presents perspectives to understand conidial germination, penetration, colonization and nutrient acquisition of the environmental form of *Fonsecaea,* and may clarify the route of infection of human skin, possibly explaining the infection route of agents of chromoblastomycosis.
